# Comparison of Muscle Onset Activation Sequences between a Golf or Tennis Swing and Common Training Exercises Using Surface Electromyography: A Pilot Study

**DOI:** 10.1155/2016/3987486

**Published:** 2016-06-15

**Authors:** John M. Vasudevan, Andrew Logan, Rebecca Shultz, Jeffrey J. Koval, Eugene Y. Roh, Michael Fredericson

**Affiliations:** ^1^Department of Physical Medicine & Rehabilitation, University of Pennsylvania, 1800 Lombard Street, Philadelphia, PA 19146, USA; ^2^Human Performance Lab, Sports Medicine Center, Department of Orthopaedic Surgery, Stanford University, 341 Galvez Street, Lower Level, Stanford, CA 94305, USA; ^3^Division of Sports Medicine, Department of Orthopaedic Surgery, Stanford University, 450 Broadway Street, Redwood City, CA 94063, USA

## Abstract

*Aim*. The purpose of this pilot study is to use surface electromyography to determine an individual athlete's typical muscle onset activation sequence when performing a golf or tennis forward swing and to use the method to assess to what degree the sequence is reproduced with common conditioning exercises and a machine designed for this purpose.* Methods*. Data for 18 healthy male subjects were collected for 15 muscles of the trunk and lower extremities. Data were filtered and processed to determine the average onset of muscle activation for each motion. A Spearman correlation estimated congruence of activation order between the swing and each exercise. Correlations of each group were pooled with 95% confidence intervals using a random effects meta-analytic strategy.* Results*. The averaged sequences differed among each athlete tested, but pooled correlations demonstrated a positive association between each exercise and the participants' natural muscle onset activation sequence.* Conclusion*. The selected training exercises and Turning Point*™* device all partially reproduced our athletes' averaged muscle onset activation sequences for both sports. The results support consideration of a larger, adequately powered study using this method to quantify to what degree each of the selected exercises is appropriate for use in both golf and tennis.

## 1. Introduction

Golf and tennis are popular sports for athletes of all ages and ability levels. There are over 30 million golfers and 28 million active tennis players in the United States and over 50 million golfers worldwide [1–4]. Both sports are associated with overuse injuries, as they rely on transferring the momentum from a club or a racquet to a ball for maximum velocity and accuracy [5].

The modern golf swing is characterized by increased shoulder rotation with restricted hip turning to maximize hip-shoulder separation. This technique may produce increased ball velocity and distance but comes at a cost of increased risk of musculoskeletal injury [5–10]. The modern golf swing has been shown to place nearly as much stress upon the spine as forces measured in college football linemen hitting a blocking sled [11]. Several biomechanical studies have assessed not only the golf swing, but also the role of axial rotation on loading and injury of the thoracolumbar spine, in which a supramaximal angle (termed the “dynamic X-factor”) takes the player beyond a safe limit of flexibility [8–14]. Control of the dynamic X-factor requires conditioning not only spinal stabilizing muscles, but all limb and trunk muscles required of the swing.

The golf swing motion is separated into several phases: backswing, forward swing, acceleration, early follow through, and late follow through. The primary lower limb and trunk muscles involved in these phases have been described [2, 5]. Although EMG studies have identified muscles active in each phase, few studies have detailed the precise order of activation in an individual athlete or have only measured a limited set of muscles (i.e., lower limb or trunk, but rarely are both regions studied at once) [8, 9, 15].

As with golf, tennis has evolved over the years to become a more aggressive sport, requiring increasing loads to maximize power. This, in turn, increases the risk of injury across all skill levels [16]. There are no longitudinal cohort studies to identify risk factors and no randomized controlled trials designed to determine methods of injury prevention.

The tennis forehand stroke is divided into the backswing and forward swing. Power in the tennis forward swing is derived from sequential contractions in the lower limbs, axial torque through the pelvis and trunk in the transverse plane, and sequential rotation through the kinetic chain to the shoulder girdle and upper limb [17, 18]. As with the golf swing, this also requires hip-shoulder separation during backswing to increase potential energy and power from the stroke. Studies sequencing muscle activation in tennis are few in number, and most focus on the serve rather than forward swing [17]. Studies comparing athletes of varying skill levels have found that appropriate timing and velocity of pelvis and trunk rotation are critical to performance in athletes of all levels [19]. However, there is little information regarding typical muscle activation patterns of the trunk and pelvis in the tennis forward swing.

Conditioning exercises specific to the golf and tennis swing have been described to maximize efficiency of movement through the kinetic chain [2, 4, 17]. Although precise reproduction of sport-specific movement may not be necessary to condition or rehabilitate athletes, exercises are often tailored to athletes to accommodate for the complex movement patterns required of their sport. The purpose of this pilot study is to use surface electromyography to determine an individual athlete's typical muscle onset activation sequence from the limbs through the trunk, with a goal of determining how well the sequence is reproduced with common conditioning exercises (wood chop cable pull, medicine ball wall throw, or the novel Turning Point 4.0 Rotational Training Platform). We sought to determine whether this electromyographic measurement method used is appropriate to analyze motions used in both golf and tennis. Understanding how well an exercise simulates an athlete's swing may provide insight into the development of an optimal and individualized exercise regimen.

## 2. Materials and Methods

### 2.1. Participants

Informed consent for participation in study was obtained from 18 healthy male subjects (9 golfers and 9 tennis players). The number of participants was kept low for the pragmatic purpose of generating and analyzing pilot data. The participants ranged from 20 to 33 (mean = 22.2) years of age. The 9 golfers ranged from 20 to 33 (22.3) years of age. All golfers had been playing regularly for 1–14 (7) years and 2 of the participants played on an intercollegiate club golf team. The 9 tennis players were between the ages of 19 and 26 (22.4). Participants for tennis had been playing tennis ranging from 6 to 15 (9.9) years. Of the 9 tennis players, 2 had played with high school varsity, 2 are currently at the division III collegiate level and 3 at the club level, and the remaining 2 participants play recreationally. In an effort to minimize variability in the swing, only male university students without acute or chronic injuries of the spine or limbs (that could interfere with club or racquet swing capability) were recruited. All subjects primarily play one of the two sports. The study protocol was approved by the Stanford University Institutional Review Board.

### 2.2. Procedures

For this study, we chose the wood chop cable pull (WC) and medicine ball wall throw (MB) exercises because strength and conditioning coaches commonly use these exercises to train and rehabilitate athletes in these sports. These two exercises in isolation are by no means comprehensive in the training of the core musculature and proper motion through the kinetic chain. Rather, they are representative of many exercises used to train rotational movement and chosen based upon the advice of the strength and conditioning coaches at our university. The Turning Point 4.0 Rotational Training Platform (Turning Point Biotechnology, Toledo, Ohio; http://www.turningpointbiotech.com/index_draft.php) (TP) was included in this study because it was not only designed specifically for training of rotational strength, but also intended to provide more consistent and measurable movement due to its adjustable settings and computerized motion analysis [20, 21].

Prior to testing, participants were given five minutes to warm up with low-intensity cardiovascular exercise (e.g., treadmill walking) or stretching to their preference. Once ready, participants had the muscles of interest prepared for EMG electrode placement by gentle abrasion of the skin and treatment with alcohol to clean the area and provide an accurate reading. Ultrasound gel was also used to ensure that a clean EMG signal was recorded for all 15 channels used in this study. Location of electrode placement on each muscle was determined according to* Cram's Introduction to Surface Electromyography* [22].

**Figure 1:**
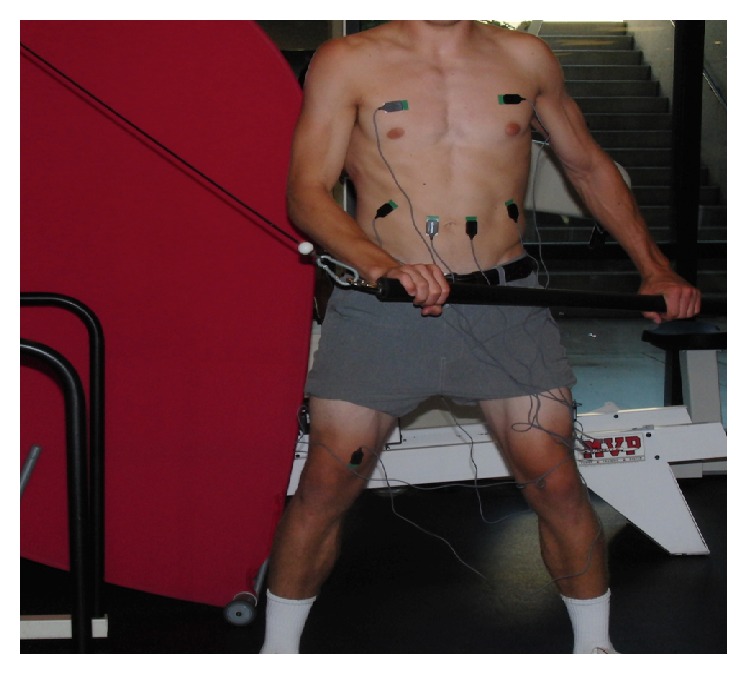
Surface EMG setup for wood chop cable pull exercise.

**Figure 2:**
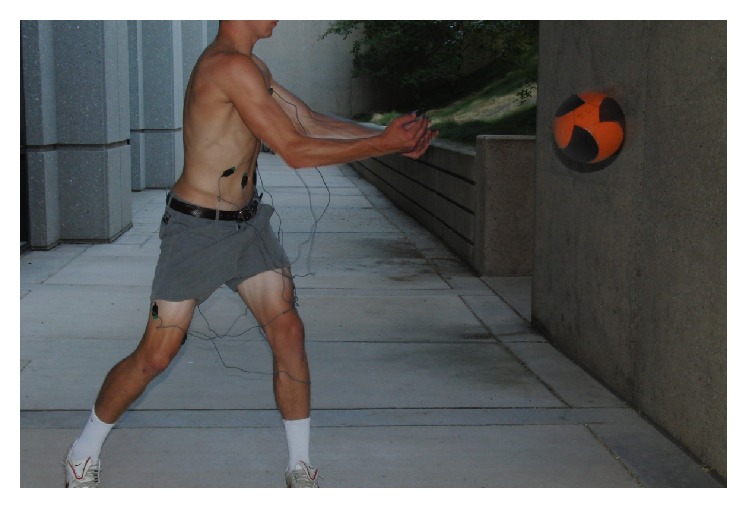
Surface EMG setup for medicine ball wall throw exercise.

**Figure 3:**
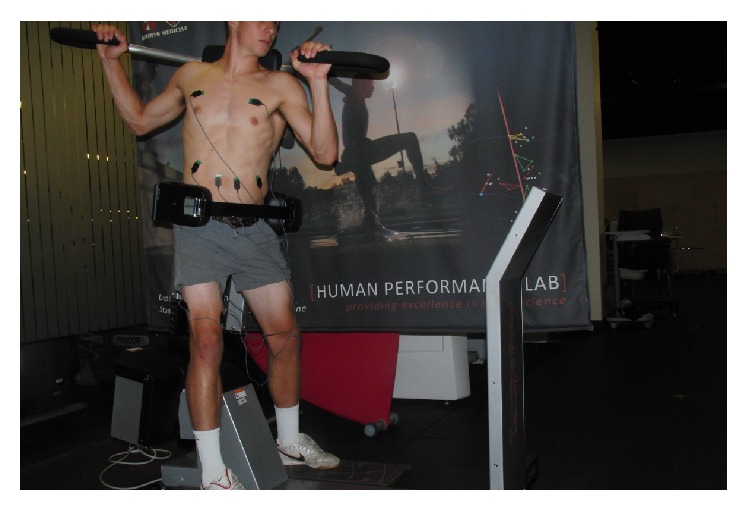
Surface EMG setup for Turning Point 4.0 Rotational Platform.

Once all EMG electrodes were properly placed, participants completed four motions, performing 5 repetitions of each—a golf or tennis forward swing with ball contact, a wood chop cable pull (Figure 1), an 8-pound medicine ball wall throw in a perpendicular stance to a wall (Figure 2), and the Turning Point 4.0 Rotational Platform (Figure 3). We chose five repetitions for each motion to sample enough data while reducing any adverse effects of fatigue as each athlete progressed through the motions. The wood chop cable pull exercise is a dynamic, functional exercise that employs the lower limbs, trunk, and upper limbs in a natural motion. The person performing the exercise moves a bar or ball in diagonal fashion (e.g., from lower right to upper left in a right-handed golfer) in a controlled, spiral movement while keeping the torso as still as possible by coactivation of abdominals and paraspinals. A medicine ball wall throw exercise can be used in similar fashion to the wood chop but allows for a more natural take away (simulating backswing) and release of the ball toward a wall (simulating follow through), since the athlete is not tethered to a machine. The Turning Point 4.0 Rotational Training Platform places the user in a standing position and allows for shoulder-hip separation required of rotational movements as used in golf and tennis.

These exercises were performed in a randomized order for each participant. Randomization was done by use of a randomized number sheet. Participants were told to complete all motions at a self-selected comfortable pace. Resistance for the WC exercise was standardized to equal the torque created by an 8-pound medicine ball. Because settings on the TP platform were not directly comparable to the other exercises, resistance on the platform for each subject was set according to an equal level of self-perceived exertion when compared to the WC and MB exercises. All exercises were performed in the individual's self-selected natural swing stance. Each participant was given up to 5 minutes to practice the motions for each exercise and the Turning Point machine, followed by 5 minutes of rest prior to recorded trials. Five minutes of rest were given between each of the four motions to control for effects of fatigue.

### 2.3. Measuring Methods

Electromyographic (EMG) data were recorded (1200 Hz) from lower limb and trunk muscles (Bagnoli Desktop, Delsys Inc., Boston. MA, USA) using preamplified single differential, rectangular Ag electrodes with 10 mm interelectrode distance (DE-2.1, Delsys, Inc., Boston, MA, USA). The 15 specific muscles were bilateral erector spinae, pectoralis major, external oblique, rectus abdominis, internal oblique, vastus lateralis, gluteus maximus, and unilateral vastus medialis of each participant's trailing leg.

EMG data were bandpass filtered at 30–500 Hz, rectified, and low-pass filtered at 30 Hz. The evaluation of EMG onset was determined with computer analysis (MATLAB version 7.10.0. Natick, Massachusetts: The MathWorks Inc., 2010). The algorithm involved identification of the time point when the mean of a specified number of surrounding time samples exceeded the mean level by a predetermined number of standard deviations. This time point is considered the onset of activation for each muscle studied. The baseline mean level was averaged for 50 ms prior to signal to perform the activity. Following this, EMG activity was averaged over a sliding time window of 25 ms. If the average of the time window did not exceed the baseline by 3 standard deviations, the sliding time window was advanced one sample at a time until the onset was found. The algorithm and parameters used have previously been demonstrated to reduce observer bias and increase reliability [23].

The muscles studied in each subject were ranked in order of activation as described above from 1 to 15. For example, if the external oblique was the first to activate, it was ranked 1, and the last muscle to activate was ranked 15. In order to account for variability among swings in each individual, this activation order was averaged across the natural swing for each subject to determine his own “gold standard.” We anticipated this variability based on a study by Meister et al. that demonstrated decreased consistency of the golf swing as handicap increases [6]. The muscle activation sequences for the wood chop cable pull (WC), medicine ball wall throw (MB), and Turning Point (TP) exercises were measured in similar fashion for comparison to each subject's natural swing.

### 2.4. Statistical Analyses

For each athlete, a Spearman correlation was calculated to estimate the congruence of muscle onset activation order between golf swing and the other exercises (i.e., WC, MB, and TP). For each exercise pair (e.g., golf swing versus WC) the correlations of each group of 9 athletes were pooled into overall estimates with 95% confidence intervals using a random effects meta-analytic strategy, implemented in the Metafor Package of the R statistical program. Meta-analysis is a statistical strategy to average or pool independent statistical results (in this case, player-specific Spearman's correlations) in a manner that retains information regarding within-player variability and number of observations [24]. Differences between the pooled correlations (e.g., golf swing versus WC compared to golf swing versus TP) were evaluated by inspecting the overlap between the 95% confidence intervals for the pooled correlations. The same method was used to estimate the congruence of muscle activation order between tennis swing and WC, MB, and TP exercises.

## 3. Results

### 3.1. Golf

**Figure 4:**
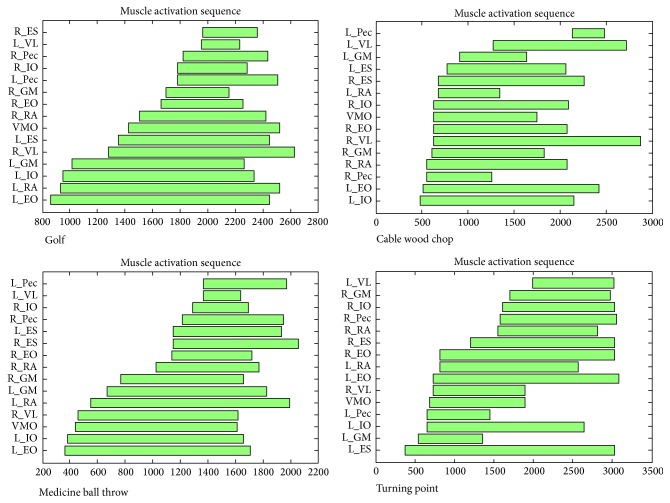
Example of one golf athlete's averaged muscle onset activation sequence for the forward golf swing, wood chop cable pull, medicine ball wall throw, and Turning Point 4.0 Rotational Platform. Each bar represents the average onset and duration of each subject's individual muscle activation. (*x*-axis: time (msec)). *y*-axis: muscles (e.g., L_EO = left external oblique).

**Figure 5:**
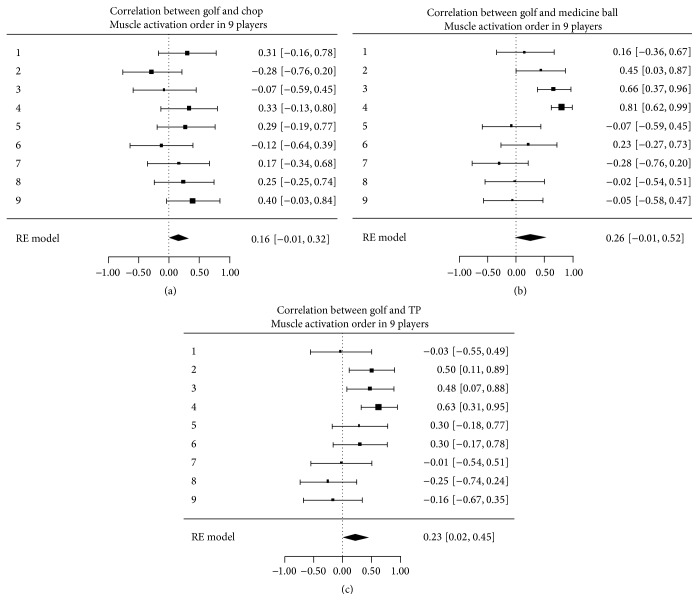
Correlation of muscle onset activation sequences between golf swing and the wood chop cable pull, medicine ball wall throw, and Turning Point 4.0 Rotational Platform. The top nine bars indicate each subject's individual correlation (with 95% confidence interval), and the bottom bar indicates the correlation averaged across all subjects tested (with 95% confidence interval).

Averaging of the 5 motions for each athlete determined the natural forehand swing for that athlete. An example of an individual athlete's muscle activation sequence for the golf swing and the three exercises studied is shown in Figure 4. There were differences in correlation (ranging from negative to positive) between the forward swing and each exercise among the 9 golf athletes, but these differences were within the range of the 95% confidence intervals. This is displayed along the *x*-axis in Figures 5(a)–5(c).

The majority of all athletes demonstrated a positive correlation between all exercises and the natural swing. For the minority of athletes in which there was a negative correlation, all confidence intervals crossed into the positive range (Figure 5, top 9 lines). When averaged among all athletes tested, the coefficients of correlation (with 95% confidence interval) between the WC, MB, and TP movements to the golf forward swing were 0.16 (95% CI: −0.01 to 0.32), 0.26 (95% CI: −0.01 to 0.52), and 0.23 (95% CI: 0.02 to 0.45), respectively (Figure 5, bottom line). The confidence intervals of these correlations overlap, indicating a nonsignificant difference among the exercises.

### 3.2. Tennis

**Figure 6:**
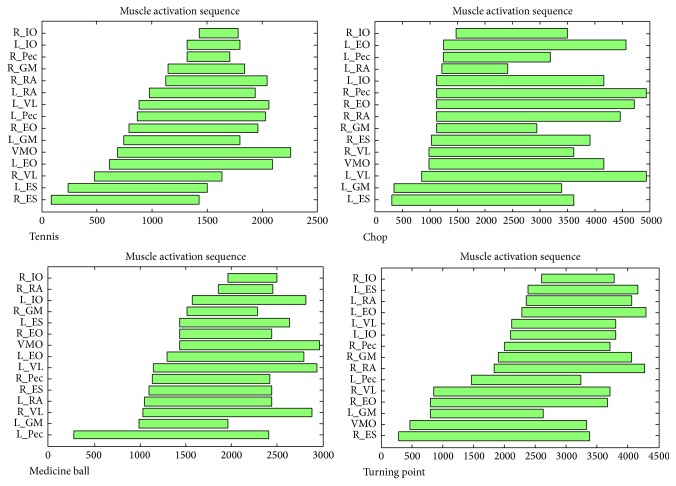
Example of one tennis athlete's averaged muscle onset activation sequence for the forehand tennis swing, wood chop cable pull, medicine ball wall throw, and Turning Point 4.0 Rotational Platform. Each bar represents the average onset and duration of each subject's individual muscle activation. (*x*-axis: time (msec)). *y*-axis: muscles (e.g., L_EO = left external oblique).

**Figure 7:**
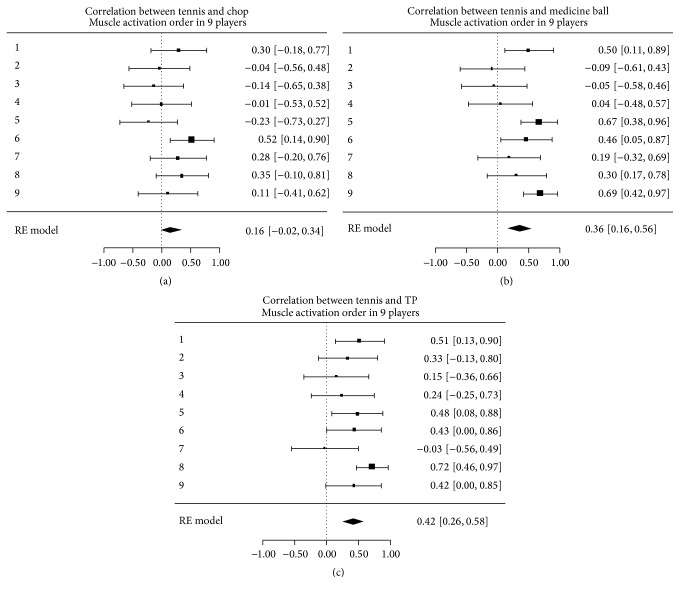
Correlation of muscle onset activation sequences between tennis swing and the wood chop cable pull, medicine ball wall throw, and Turning Point 4.0 Rotational Platform. The top nine bars indicate each subject's individual correlation (with 95% confidence interval), and the bottom bar indicates the correlation averaged across all subjects tested (with 95% confidence interval).

An example of an individual athlete's muscle activation sequence for the tennis swing and the three exercises studied is shown in Figure 6, using the same methodology as with the golf athletes. Again, there were differences in correlation (ranging from negative to positive) between the forward swing and each exercise among the 9 tennis athletes, with these differences within the range of the 95% confidence intervals. This is displayed along the *x*-axis in Figures 7(a)–7(c). This particular group studied exhibited less inconsistency than the golf athletes.

As with the golf cohort, the majority of all athletes demonstrated a positive correlation between all exercises and the natural swing. For the minority of athletes, in which there was a negative correlation, confidence intervals crossed into the positive range (Figure 7, top 9 lines). When averaged across all participants, the coefficients for WC, MB, and TP to the tennis forward swing were 0.16 (−0.02–0.34), 0.36 (0.16–0.56), and 0.42 (0.26–0.58), respectively (Figure 7, bottom line). The confidence intervals of these correlations overlap, indicating a nonsignificant difference among the exercises.

## 4. Discussion

This study utilized surface electromyography to observe the muscle activation sequences among recreational and club-level golf and tennis athletes. Seminal studies by Perry and colleagues, such as the ones by Pink et al. in 1990 and 1993 and Watkins et al. in 1996, helped establish the use of electromyography to evaluate muscle activation in the golf swing [25–27]. Because most of the available data is focused on trunk and shoulder musculature, recent reviews of electromyography variables related to the golf swing have identified a gap in the knowledge of lower limb muscle activation [28]. Based on what was already known (and yet unknown), the purpose of this pilot study was to not only define a method to readily identify the precise onset sequence of the lower limb through the trunk muscles in an athlete of any level, but also apply the method across multiple sport motions (e.g., golf and tennis forward swing).

Conclusions from the study data are limited by small sample size but suggest that the selected exercises may be appropriate for both golf and tennis. A larger, adequately-powered study would generate sufficient data to quantify the value of these exercises with greater certainty. The results of this study indicate that, when results are averaged across all athletes studied, there are positive correlations between the selected exercises and the natural forward golf and tennis swing in the population studied. The TP more closely correlates to the tennis swing than golf swing in the subjects tested, but not to a significant degree when compared to the other exercises studied. We suspect that the TP may have an advantage in tennis as the sport features more rotation in the transverse plane, of which the majority of the TP device's motion pertains. The golf swing not only employs transverse trunk motion, but also employs dynamic lateral trunk bending. While the TP device allows the user to set the trunk angle, this angle remains static throughout the motion of the device. Conversely, the tennis forward swing (as opposed to the overhead swing or serve) requires less dynamic change in trunk angle. These conclusions are made with caution, however, due to the small sample size. Because the confidence intervals overlapped among all motions tested, there was no demonstrable advantage to any one exercise.

A question for future study is to determine what degree of inconsistency or variability in sport motion is acceptable in order to achieve the desired performance outcome. Fleisig et al. studied the variability of pitching biomechanics from amateur to elite levels, and while professional athletes demonstrated less variability in their motions than their amateur counterparts, differences in temporal and kinetic variation were nonsignificant [29]. Wagner et al. found similar decreases in variability in certain motions in more highly skilled team handball players, but also they observed that athletes had an ability to maintain accuracy despite increases in movement variability required by the circumstances of the game [30]. The implication of these studies, as well as similar observations by Bartlett et al., is that a certain amount of variability may be desirable and functional without adversely affecting the sport task outcome or injury risk [31].

The most significant limitation of this study is the small sample size. However, the intention was to use a convenience sample size to develop pilot data that would promote use of this method in future studies. Another limitation is our* a priori* assertion that the selected exercises are effective at reproducing a typical muscle activation sequence. No single exercise will perfectly simulate the natural swing, and a larger combination of exercises (whether they are intended to mimic the sport motion or not) is necessary to improve performance.

Movement patterns will vary not only by sport, but also by variations in the competitive environment [32]. Furthermore, muscle activation may vary in the context of recent injury [33]. Electromyographic methods have been used to measure the activation sequences in sport, but this is the first to our knowledge specifically studying the average muscle onset sequence from the lower limb through the trunk (distal-to-proximal kinetic chain) in the golf and tennis forward swing. The method used in this study has the potential for use in future studies to measure the precise timing of muscle onset activation in other sports and contexts (e.g., after injury) as well. The use of this method in future studies will allow for more significant conclusions to be drawn when examining the relationship of one exercise compared to another for the purposes of training and, eventually, determining benchmarks for return-to-play during injury rehabilitation.

## 5. Conclusions

Using surface electromyography to record the average onset muscle activation sequence of lower limb and trunk muscles in recreational and club-level golf and tennis athletes, there was a positive average correlation of the cable pull wood chop, medicine ball wall throw, and Turning Point 4.0 Rotational Platform to the natural golf and tennis forward swing in the amateur athlete population studied. Our pilot data suggest that a larger study using this method may effectively quantify to what degree these and other exercises are appropriate for golf and tennis athletes.
